# Association of Traumatic Injury and Incident Myocardial Infarction and Stroke: A Prospective Population-Based Cohort Study

**DOI:** 10.31083/j.rcm2405136

**Published:** 2023-04-28

**Authors:** Xin Liu, Aitian Wang, Tao Liu, Yue Li, Shuohua Chen, Shouling Wu, Haojun Fan, Jingli Gao, Xiaolan Li, Shike Hou, Chunxia Cao

**Affiliations:** ^1^Institute of Disaster and Emergency Medicine, Tianjin University, 300072 Tianjin, China; ^2^Department of Intensive Medicine, Kailuan General Hospital, 063001 Tangshan, Hebei, China; ^3^Department of Cardiology, Kailuan General Hospital, 063001 Tangshan, Hebei, China

**Keywords:** traumatic injury, myocardial infarction, stroke, cohort study

## Abstract

**Background:**

Several studies have linked traumatic injury and cardiovascular disease. However, few studies have investigated the associations between traumatic injury and cardiovascular disease subtypes. We aimed to prospectively examine the association between traumatic injury and the risk of incident myocardial infarction (MI) and stroke.

**Methods:**

This study was based on a prospective cohort study that included 13,973 patients who had been hospitalized for traumatic injuries from 1980 to 2020. We randomly selected 4 uninjured participants from the cohort study for each patient as controls matched by age (±3 years) and sex. All participants were free of MI and stroke at enrollment. Cox regression was used to examine the association between traumatic injury and incident MI and stroke.

**Results:**

During a median follow-up period of 13.5 years, 1032 cases of MI and 4068 cases of stroke were recorded. After multivariable adjustment, relative to controls, patients with severe injury had the highest hazard ratio (HR) for MI (HR = 1.93; 95% CI: 1.26–2.96) and stroke (HR = 1.60; 95% CI: 1.25–2.05). The HRs of MI and stroke were 0.97 (0.81–1.17) and 1.11 (1.02–1.21) for patients with mild injury and 1.28 (0.97–1.69) and 1.22 (1.06 to 1.41) for patients with moderate injury. Additionally, patients with older age at injury and chest injury had a higher HR for MI and stroke (*p*-interaction < 0.05).

**Conclusions:**

Traumatic injury appears to be associated with an increased risk of incident MI and stroke. Therefore, early screening and prevention of MI and stroke following a traumatic injury are needed.

## 1. Introduction

Cardiovascular disease (CVD) is the leading cause of death worldwide [1], with myocardial infarction (MI) and stroke being the most prevalent and major causes of CVD-related mortality [2, 3]. Although behavioral and biological risk factors were mainly associated with developing CVD [4], recent studies have shown that traumatic injury may influence the risk of developing CVD [5, 6]. Traumatic injury is a major public health concern, with cases widely distributed among all age groups [7]. Injured patients tend to develop negative coping behaviors following injury, which can damage physical health. In fact, traumatic injury was associated with multiple behavioral CVD risk factors, such as smoking, substance abuse, and physical inactivity [8, 9]. The association between traumatic injury and CVD risk factors has prompted an increasing interest in exploring the association between traumatic injury and CVD-related health outcomes.

Several studies have linked traumatic injury to the surge in CVD events [10, 11]. A survey from Canada found spinal cord injury to be associated with significantly increased odds of CVD [12]. Similarly, a study from Hong Kong showed that the risk of CVD in patients with hip fractures increased by 27% more than in propensity score-matched controls [13]. Previous research has usually classified all cardiovascular disease events into one group and few studies have prospectively investigated the associations between traumatic injury and the subtypes of CVD events (MI and stroke). Therefore, the objective of this study was, in a prospective cohort study, to examine the associations between traumatic injury and the risk of incident MI and stroke.

## 2. Materials and Methods

### 2.1 Study Design and Study Population

This study was based on the Kailuan Study, a prospective cohort study conducted in the Kailuan community in Tangshan, China. The detailed study design and procedures have been described previously [14, 15]. Briefly, the Kailuan Study conducted the first health survey enrolling on-job and retired employees of the Kailuan Group from July 2006 to October 2007 [14, 15]. Participants responded to questionnaires and underwent health examinations in 11 local hospitals. Data on demographics, lifestyle, basic anthropometric measurement, and blood tests, were gathered and subsequently updated every 2 years. From July 2006 to October 2019, a total of 171,089 participants at least 18 years old were enrolled in the Kailuan Study.

In this study, individuals from the Kailuan study who had been hospitalized for traumatic injuries from January 1980 to December 2020 were included. An expert panel was made up of three experienced clinicians from the emergency department, orthopedics and general surgery checked the admission and discharge lists annually from the 11 local hospitals and the social insurance records to ascertain these injured patients. The panel defined injury severity (mild, moderate, and severe injury) according to the injury conditions of patients at the time of admission. The classification criteria were as follows. First, the mild injury was defined as no injury in the main sites and organs (e.g., brain, thoracic cavity, and abdominal cavity) of the patient, mostly skin and soft tissue injury or fracture of the distal limb. Second, the moderate injury was defined as an injury to the main sites or organs of the patient with relatively stable vital signs and no short-term life-threatening events (e.g., abdominal organ injury and long bone fracture). Third, severe injury was defined as an injury to the main sites or organs of the patient, if not a timely and effective treatment, which can lead to death in a short time (e.g., severe bleeding and several fractures of the skull vault). Patients with repeated injuries were classified according to the heavier grade of injury [16]. We excluded participants with missing information on age and sex and a history of MI or stroke at enrolment in the Kailuan Study (**Supplementary Fig. 1**). For each patient, four uninjured participants of the Kailuan Study were randomly selected as controls matched by age (±3 years) and sex. The followed-up was started at the time of admission for injured patients and the same day for matched controls, and until the diagnosis of incident MI or stroke, death, or 31 December 2020. Ultimately, 13,973 injured patients and 55,892 controls were included in the study.

This study followed the principles expressed in the Declaration of Helsinki. The ethics committee of Kailuan General Hospital approved the study protocol (Approval number: 2006-05). All participants provided written informed consent and did not receive financial compensation.

### 2.2 Ascertainment of Outcome Events

The outcome event in this study was the first incident of MI or stroke (no history of outcome events before the start of follow-up and for multiple occurrences of MI or stroke, follow-up ended at the first visit) during the follow-up period. All outcome events were identified according to the International Classification of Diseases 10th revision (ICD-10, MI: I21, I60 and I61; stroke: I63) [17]. Information on MI or stroke was obtained from the Hospital Discharge Register of 11 location hospitals and from the Municipal Social Insurance Institution, and updated annually during the follow-up period. Suspected cases of MI or stroke were identified by a review of annual discharge records by three experienced clinicians from the Department of Cardiology. The diagnosis of MI was determined by the patient’s clinical symptoms, electrocardiogram, and dynamic changes in myocardial enzymes, following the World Health Organization’s Multinational Monitoring of Trends and Determinants in Cardiovascular Disease criteria [18]. The diagnosis of stroke was based on neurological signs, clinical symptoms, and neuroimaging tests (including computed tomography and magnetic resonance imaging), in line with the World Health Organization criteria [19]. The information of all-cause mortality was from the municipal death registries. and checked annually against local residential records, with active survival confirmation through subdistrict offices.

### 2.3 Assessment of Covariates

Covariates in this study were gathered from the self-reported questionnaires (including age, sex, smoking and drinking status, physical activity, salt intake, and family history of MI and stroke), basic anthropometric measurements (including height, weight, systolic blood pressure [SBP], and diastolic blood pressure [DBP]), and blood tests (including fasting blood glucose [FBG], low-density lipoprotein cholesterol [LDL-c], high-density lipoprotein cholesterol [HDL-c] and triglycerides [TG]) [20, 21, 22, 23]. All covariates were collected when participants enrolled in the Kailuan Study.

Body mass index (BMI) is calculated by dividing weight (kg) by height squared ($m^{2}$). BMI ≥28.0 kg/$m^{2}$ was defined as obesity according to the Working Group on Obesity in China guidelines [24]. Smoking and drinking statuses were classified as non-drinker or non-smoker and current smoker or drinker, according to the self-reported questionnaires. Physical activity was classified as inactive or active, according to the frequency (whether ≥4 times/week and ≥20 min/time) during leisure time. According to whether daily habitual salt intake is ≥12 g, salt intake was classified as high salt intake and non-high salt intake [25].

The blood tests at each health assessment were conducted after overnight fasting. The blood samples were analyzed using an auto-analyzer (Hitachi 747, Hitachi, Tokyo, Japan) at the central laboratory of Kailuan General Hospital [26].

### 2.4 Statistical Analysis

The baseline characteristics were compared between injured patients and controls. Variance or the Kruskal-Wallis test was used for continuous variables, and the chi-square test was used for categorical variables. The incidence rate of MI and stroke per 1000 person-years was calculated by the total number of incident cases divided by the total number of years of follow-up and multiplied by 1000. Cox proportional hazards models were used to calculate the hazard ratio (HR) and 95% confidence intervals (CI) of incident MI and stroke. Covariates with *p *< 0.2 were adjusted in multivariable models, and the log (–log survival) plots were used to assess the proportional hazards assumption [27, 28].

Furthermore, two stratified analyses were conducted to determine the role of age at injury and sites of injury in the association between traumatic injury and incident MI or stroke. Patients with multiple injuries were classified based on the major site of injury. Each control was assigned a false age at injury and a site of injury similar to those of the matched injured patient. The likelihood ratio test was used to examine the interaction between traumatic injury and age at injury and sites of injury.

Several sensitivity analyses were conducted to test the robustness of our findings. (1) The MI and stroke events incident within the first year of the follow-up periods were excluded to minimize potential reverse causation. (2) Injured patients who had been injured for >30 years and (3) those who had been injured for <5 years were excluded to reduce the effects of length of time after traumatic injury. In addition, two subgroup analyses of (4) men and (5) women were conducted, respectively, since there were far more men than women in this study.

All analyses were performed using SAS (version 9.4, SAS Institute, Cary, NC, USA). All statistical tests were two-sided, and *p *< 0.05 was accepted as statistically significant.

## 3. Results

### 3.1 Baseline Characteristics

The baseline characteristics of 13,973 injured patients and 55,892 controls were shown in Table 1. In general, the mean age of participants was 52.3 ± 9.3 years, and 4.5% of the participants were women. Of 13,973 injured patients, 10,259 (73.4%) had mild injuries. In comparison with the control, injured patients were more likely to be drinkers or smokers, had a family history of MI or stroke, and were less likely to engage in active physical activity. Furthermore, injured patients had lower TG, FBG, LDL-c, SBP, and DBP levels than controls. Additionally, with increasing injury severity, the proportions of smokers, drinkers, and patients with a family history of MI or stroke decreased, whereas the proportions of patients engaging in active physical activity and having a higher age, FBG, SBP, and DBP increased (**Supplementary Table 1**).

**Table 1.** **Descriptive statistics of injured and uninjured subjects**.

Variables	Total	Uninjured	Injured	*p* value
	(N = 69,865)	(N = 55,892)	(N = 13,973)	
Age, yrs	52.3 ± 9.3	52.3 ± 9.3	52.3 ± 9.3	0.989
Male	66,690 (95.5)	53,352 (95.5)	13,338 (95.5)	1.000
Active physical activity	10,419 (14.9)	8410 (15.0)	2009 (14.4)	0.047
Drinker	13,956 (20.0)	10,767 (19.3)	3189 (22.8)	<0.001
Smoker	30,871 (44.2)	24,597 (44.0)	6274 (44.9)	0.057
High salt intake	7713 (11.0)	6153 (11.0)	1560 (11.2)	0.600
Family history	3138 (4.5)	2364 (4.2)	774 (5.5)	<0.001
Obesity	11,805 (16.9)	9416 (16.8)	2389 (17.1)	0.480
TG, mmol/L	1.8 ± 1.5	1.8 ± 1.5	1.7 ± 1.4	0.004
FBG, mmol/L	5.6 ± 1.7	5.7 ± 1.7	5.5 ± 1.6	<0.001
HDL-c, mmol/L	1.5 (1.2–1.7)	1.5 (1.2–1.7)	1.5 (1.3–1.8)	<0.001
LDL-c, mmol/L	2.5 (2.0–3.0)	2.5 (2.0–3.0)	2.4 (1.9–3.0)	<0.001
SBP, mmHg	132.5 ± 20.2	132.7 ± 20.2	131.8 ± 20.3	<0.001
DBP, mmHg	85.0 ± 11.7	85.0 ± 11.7	84.8 ± 11.6	0.068

Data are presented as mean ± standard deviation, median (interquartile range), or N (%). Abbreviations: yrs, years; TG, triglycerides; FBG, Fasting Blood Glucose; HDL-c, high-density lipoprotein cholesterol; LDL-c, low-density lipoprotein cholesterol; SBP, systolic blood pressure; DBP, diastolic blood pressure.

### 3.2 Risk of MI and Stroke

**Fig. 1.**
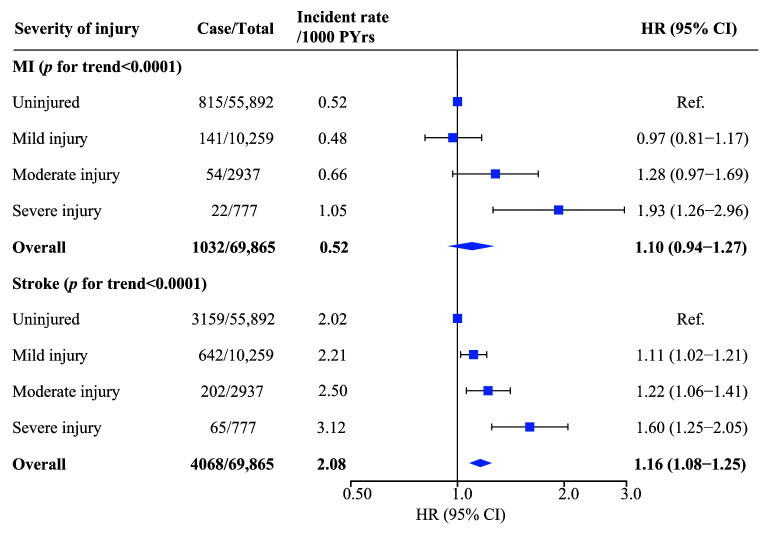
**Adjusted HR (95% CI) of incident MI and stroke for injured patients according to injury severity**. Abbreviations: MI, myocardial infarction; PYrs, person-years; HR, hazard ratio; CI, confidence interval.

The median follow-up period for participants was 13.5 (8.6–14.1) years. During the period, 1032 cases of MI and 4068 cases of stroke were identified. The median time of developed as MI or stroke after injury was 5.68 (2.93–7.95) years and 7.51 (4.14–10.10) years, respectively. Fig. 1 shows the incidence rates of MI and stroke per 1000 person-years and the HR and 95% CI of incident MI and stroke among injured patients. Patients with severe injury have a higher incidence rate per 1000 person-years of MI and stroke (1.05 and 3.12 per 1000 person-years, respectively). After multivariable adjustment, we observed a significant trend of increasing risks of MI and stroke with injury severity among injured patients (*p*-trend = 0.0128 for MI and <0.0001 for stroke). Compared to controls, patients with severe injury had the highest risk for MI and stroke, and 93% and 60% of these patients were more likely to develop MI and stroke, respectively (HRs [95% CI] = 1.93 [1.26–2.96] and 1.60 [1.25–2.05], respectively). No evidence of an association between mild/moderate injury and increased risk of MI (HRs [95% CI] = 0.97 [0.81–1.17] and 1.28 [0.97–1.69], respectively) was present. For patients with mild/moderate injury, HRs (95% CI) for stroke were 1.11 (1.02–1.22) and 1.22 (1.06–1.41), respectively.

### 3.3 Stratified Analyses by Age at Injury and Sites of Injury

In the stratified analyses, injured patients and controls were classified into four groups according to age at injury: <30 years at injury, 30–39 years at injury, 40–49 years at injury, and ≥50 years at injury (Table 2). Based on the injury site, they were classified into five groups: head, chest, abdominal, extremity, and other site injuries (Table 3). After multivariable adjustment, the association between traumatic injury and risk of incident MI and stroke differed by age at injury and sites of injury, compared to controls (*p*-interaction < 0.0001 and = 0.0456 for MI; <0.0001 and <0.0001 for stroke, respectively). Notably, the risks of incident MI and stroke gradually increased with age at injury. Patients with ages ≥50 years at injury had the highest risk for MI and stroke (HRs [95% CI] = 2.58 [1.73–3.85] and 2.24 [1.78–2.81], respectively, Table 2). Additionally, patients with chest injuries had a higher risk of incident MI (HR [95% CI] = 1.85 [1.22–2.80]), and patients with chest injuries or extremity injuries had a higher risk of incident stroke (HRs [95% CI] = 1.82 [1.46–2.28] and 1.12 [1.01–1.23], respectively).

**Table 2.** **Adjusted HR (95% CI) of MI and stroke for injured patients according to age at injury**.

Age at injury		Uninjured controls		Injured patients		HR (95% CI)
		Case/Total	Incident rate/1000 PYrs	Case/Total	Incident rate/1000 PYrs	
MI (*p* for interaction < 0.0001)						
	<30 yrs	215/18,116	0.39	56/4529	0.34	0.73 (0.54–0.98)
	30–39 yrs	236/16,908	0.49	65/4227	0.53	1.20 (0.91–1.58)
	40–49 yrs	260/15,652	0.64	62/3913	0.74	1.50 (1.13–1.99)**
	≥50 yrs	104/5216	0.78	34/1304	1.60	2.58 (1.73–3.85)***
Stroke (*p* for interaction < 0.0001)						
	<30 yrs	848/18,116	1.54	272/4529	1.64	0.88 (0.77–1.01)
	30–39 yrs	906/16,908	1.90	257/4227	2.10	1.24 (1.08–1.43)*
	40–49 yrs	981/15,652	2.42	272/3913	3.27	1.95 (1.69–2.25)***
	≥50 yrs	424/5216	3.21	108/1304	5.17	2.24 (1.78–2.81)***

*p < 0.05, **p < 0.01, and ***p < 0.001. Abbreviations: yrs, years; MI, myocardial infarction; PYrs, person-years; HR, hazard ratio; CI, confidence interval.

**Table 3.** **Adjusted HR (95% CI) of MI and stroke for injured patients according to sites of injury**.

Sites of injury		Uninjured controls		Injured patients		HR (95% CI)
		Case/Total	Incident rate/1000 PYrs	Case/Total	Incident rate/1000 PYrs	Case/Total
MI (*p* for interaction = 0.0456)						
	Head injury	33/2496	0.48	10/624	0.59	1.30 (0.64–2.66)
	Chest injury	93/4832	0.71	31/1208	1.17	1.85 (1.22–2.80)**
	Abdominal injury	74/5004	0.53	23/1251	0.66	1.31 (0.82–2.10)
	Extremities injury	465/33,868	0.48	112/8467	0.45	0.95 (0.77–1.16)
	Other injury	150/9692	0.55	41/2423	0.59	1.09 (0.77–1.55)
Stroke (*p* for interaction < 0.0001)						
	Head injury	145/2496	2.13	41/624	2.44	1.22 (0.86–1.73)
	Chest injury	337/4832	2.60	107/1208	4.10	1.82 (1.46–2.28)***
	Abdominal injury	267/5004	1.93	80/1251	2.33	1.26 (0.98–1.62)
	Extremities injury	1,851/33,868	1.94	538/8467	2.19	1.12 (1.01–1.23)*
	Other injury	559/9692	2.06	143/2423	2.05	1.02 (0.85–1.22)

*p < 0.05, **p < 0.01, and ***p < 0.001. Abbreviations: yrs, years; MI, myocardial infarction; PYrs, person-years; HR, hazard ratio; CI, confidence interval.

### 3.4 Sensitivity Analyses

The result of sensitivity analyses excluding MI and stroke events incident within the first year of follow-up (N = 69,443), patients with traumatic injury who had been injured for >30 years at baseline (N = 59,321), those who had been injured for <5 years (N = 57,916), and the result of the subgroup analyses in man and women (N = 66,690) were shown in **Supplementary Tables 2–6**.

## 4. Discussion

In this study, traumatic injury was associated with an increased risk of MI and stroke during a median follow-up period of 13.5 years. Notably, the risks of incident MI and stroke increased with injury severity. Additionally, patients with older age at injury or a chest injury have higher HRs of MI and stroke.

MI and stroke are the leading causes of mortality worldwide [29]. Chiang *et al*. [30] reported a 29% increased risk of MI among 8758 patients with hip fractures during a median follow-up period of 3.2 years. Variably, a significant association only existed between severe traumatic injury and incident MI among patients during a median follow-up period of 13.5 years. Some findings may explain the difference. This finding may be due to decreasing risk of MI over time after traumatic injury [5, 13]. The study showed that the risk of MI persists for >10 years following severe injury and suggested that increased clinical attention should be paid to the long-term risk of MI among patients with severe injury. A study from Taiwan reported a 2.85-fold risk of stroke within 4 years after injury among 2806 patients with spinal cord injury [31]. The risk still increased by 29% within 10 years after injury, as reported by Danish [5]. The risk in these findings was higher than the risk of 16% estimated in our study. Nevertheless, Our results are consistent with the findings of previous studies. Additionally, our result extended these findings by showing the risk of incident MI and stroke among patients was increased with injury severity.

Furthermore, the association between traumatic injury and incident MI and stroke differed by age at injury and injury site. Age effects reflect the biological and social processes of aging intrinsic to individuals [32]. Therefore, differences in metabolic condition and recovery of injured patients exist at different ages of injury. Clinical recovery was worse, and the burden of complications was greater with the increasing age of injury [33]. Ismailov *et al*. [34] reported that abdominal or pelvic traumatic injury was associated with a 65% and 93% increased risk of acute MI among patients with ages of ≤45 and ≥46 years at injury, respectively. Although patients with ages <30 years at injury had slightly better outcomes than controls, the risks of MI and stroke increased with age at injury. Additionally, incident MI was strongly associated with chest injuries, and incident stroke was strongly associated with chest and extremity injuries. A retrospective cohort study found that head and neck injuries were associated with a higher incidence of stroke after adjusting for demographics and trauma severity [35]. Although differences were observed in the control group, the incidences of MI and stroke per 1000 person-years among patients with head injury were not significantly higher than those of patients with other sites of injury. Further studies are needed to investigate the specific risks of MI and stroke among patients with different sites of injury. However, the risks of MI and stroke undoubtedly differed by the site of injury.

This study provides strong evidence linking traumatic injury with incident MI and stroke. Although the pathogenesis underlying this association could not be elucidated, some mechanisms may explain this association. First, traumatic injury triggers a range of host responses, including immune, endocrine, and inflammatory responses [6]. Existing evidence suggests that traumatic injury induces neuroendocrine activation of the hypothalamic-pituitary-adrenal axis and the sympathetic nervous system as the core [36]. However, these responses are consistently activated in injured patients and result in impairment of metabolism and cardiovascular function [37, 38]. Traumatic injury produces an excess of pro-inflammatory mediators, leading to systemic inflammatory responses [39]. A meta-analysis showed significantly high levels of inflammatory markers, including C-reactive protein, interleukin-6, and tumor necrosis factor-α, in trauma patients [40]. These inflammatory markers are associated with a higher incidence rate of MI and stroke [41]. More specifically, C-reactive protein is involved in mediating the association between traumatic injury and CVD events [42]. Additionally, traumatic injury has been associated with a propensity toward several adverse health behaviors, such as drinking, smoking, and being sedentary [43, 44]. These adverse health behaviors increase the risk of CVD events among injured patients. Moreover, injured patients have increased vulnerability towards developing mental health problems and living with multiple overlapping psychiatric disorders, including depression, anxiety, and post-traumatic stress disorder [45], which may lead to the incidence of CVD.

This study had some limitations. First, mental conditions such as poor mood, depression, and anxiety in post-injury patients were not considered, all of which are associated with traumatic injury and could explain the study results. Second, we lack data on the recovery status of injured patients, and patients who have not fully recovered may be at greater risk for MI and stroke due to physical activity limitations than those who have fully recovered, which is more pronounced in patients with severe injuries, and therefore we may be underestimating the risk in these not fully recovered patients. Additionally, since the Kailuan Study was based predominantly on men and the incidence of traumatic injury is much higher in Chinese men than in women, the majority of participants in this study were men [46]. In the female subgroup, we did not find a significant association between traumatic injury and the incident of MI and stroke. The 95% CI of HR was wider, which may be related to fewer cases in women. Thus further studies with equal sex distribution are still warranted.

## 5. Conclusions

Traumatic injury was associated with an increased risk of MI and stroke. The incidence of traumatic injury is still surging, possibly translating into a high disease burden of MI and stroke in the future. Therefore, strategies for early identification and prevention of post-traumatic MI and stroke, which are essential for injured patients improving the quality of life, should be developed.

## Data Availability

The data presented in this study are available on request from the corresponding author. The data are not publicly available due to privacy.

## Supplementary Material

Supplementary material associated with this article can be found, in the online version, at https://doi.org/10.31083/j.rcm2405136.
