# Supplementary material for: Association of Traumatic Injury and Incident Myocardial Infarction and Stroke: A Prospective Population-Based Cohort Study
Source: Rev Cardiovasc Med. 2023 Apr 28;24(5):136. doi: 10.31083/j.rcm2405136 (PMC11273029; doi:10.31083/j.rcm2405136)
Supplement: Supplementary file 1 [file 2153-8174-24-5-136-s1.zip › 2153-8174-24-5-136-s1.docx]

# Supplemental Information

**Association of traumatic injury and incident myocardial infarction and stroke: a prospective population-based cohort study**

# Supplemental Information:

**Figure** **S1** Eligibility of the study participants.

**Table S1** Descriptive statistics of injured subjects by injury severity and uninjured subjects.

**Table S2** Sensitivity analysis excluding incident MI and stroke events within the first year of the follow-up period (N = 69,443).

**Table S3** Sensitivity analysis excluding injured patients who had been injured for more than 30 years at baseline (N = 59,321).

**Table S4** Sensitivity analysis excluding injured participants who had been injured for less than 5 years (N = 57,916).

**Table S5** Sensitivity analysis in men subgroup (N = 66,690).

**Table S6** Sensitivity analysis in women subgroup (N = 3,175).

Prospective cohort Study participants between July 2006 and October 2019 (N = 171,089).

5,903 Excluded

2,428 Missing age or sex information

3,475 Had a history of MI and stroke at the enrollment

~~m~~

165,186 Participants met the excluded criteria

13,973 Participants with a traumatic injury diagnosis in 11 local hospitals

151,213 Participants without a traumatic injury diagnosis in 11 local hospitals

Randomly selected 4 uninjured participants as the controls matched by age (± 3 years) and sex in Kailuan Study

13,973 injured patients and 55,892 controls were included in the study.

**Figure S1** Eligibility of the study participants.

**Table S1** Descriptive statistics of injured subjects by injury severity and uninjured subjects

| **Variables** | **Uninjured**  **(N=55,892)** | **Mild injury (N=10,259)** | **Moderate injury**  **(N=2,937)** | **Severe injury**  **(N=777)** | ***P* value** |
| --- | --- | --- | --- | --- | --- |
| **Age, yrs** | 52.3 ± 9.3 | 51.5 ± 9.2 | 53.7 ± 8.8 | 58.5 ± 9.6 | <.0001 |
| **Male,** N (%) | 53,352 (95.5) | 9,788 (95.4) | 2,794 (95.1) | 756 (97.3) | 0.0772 |
| **Active physical activity,** N (%) | 8,410 (15.0) | 1,339 (13.1) | 503 (17.1) | 167 (21.5) | <.0001 |
| **Drinker,** N (%) | 10,767 (19.3) | 2,417 (23.6) | 629 (21.4) | 143 (18.4) | <.0001 |
| **Smoker,** N (%) | 24,597 (44.0) | 4,676 (45.6) | 1,290 (43.9) | 308 (39.6) | 0.0015 |
| **High salt intake,** N (%) | 6,153 (11.0) | 1,171 (11.4) | 310 (10.6) | 79 (10.2) | 0.4193 |
| **Family history** | 2,364 (4.23) | 587 (5.27) | 159 (5.41) | 28 (3.60) | <0.0001 |
| **Obesity,** N (%) | 9,416 (16.8) | 1,733 (16.9) | 514 (17.5) | 142 (18.3) | 0.5903 |
| **TG,** mmol/L | 1.8 ± 1.5 | 1.7 ± 1.4 | 1.7 ± 1.4 | 1.7 ± 1.3 | 0.0357 |
| **FBG,** mmol/L | 5.7 ± 1.7 | 5.5 ± 1.6 | 5.5 ± 1.6 | 5.7 ± 1.9 | <.0001 |
| **HDL-c,** mmol/L | 1.5 (1.2–1.7) | 1.5 (1.3–1.8) | 1.5 (1.3–1.8) | 1.5 (1.2–1.7) | <.0001 |
| **LDL-c,** mmol/L | 2.5 (2.0–3.0) | 2.5 (2.0–2.9) | 2.4 (1.9–3.0) | 2.4 (1.8–3.0) | <.0001 |
| **SBP,** mmHg | 132.7 ± 20.2 | 131.1 ± 19.9 | 133.0 ± 20.9 | 135.8 ± 21.4 | <.0001 |
| **DBP,** mmHg | 85.0 ± 11.7 | 84.6 ± 11.5 | 85.4 ± 12.0 | 85.7 ± 11.8 | 0.0002 |

Statistical tests performed: variance, Kruskal-Wallis test or chi-square test. Data are presented as mean ± standard deviation, median (interquartile range), or N (%). Abbreviations: yrs, years; TG, triglycerides; FBG, fasting blood glucose; HDL-c, high-density lipoprotein cholesterol; LDL-c, low-density lipoprotein cholesterol; SBP, systolic blood pressure; DBP, diastolic blood pressure;

**Table S2 Sensitivity analysis excluding incident MI and stroke events within the first year of the follow-up period (N = 69,443).**

|  | **Cases/Total** | **Incident rate/1000PYrs** | **Multivariate adjusted model** | ***P* for trend** |
| --- | --- | --- | --- | --- |
| **MI** | | | | |
| Uninjured | 701/55,539 | 0.45 | Ref. | 0.0092 |
| Mild injury | 128/10,219 | 0.44 | 1.02 (0.85-1.23) |  |
| Moderate injury | 46/2,917 | 0.57 | 1.27 (0.95-1.72) |  |
| Severe injury | 19/768 | 0.91 | 2.03 (1.28-3.21) |  |
| Overall |  |  | 1.13 (0.96-1.33) |  |
| **Stroke** | | | | |
| Uninjured | 2,901/55,539 | 1.87 | Ref. | <0.0001 |
| Mild injury | 611/10,219 | 2.11 | 1.15 (1.05-1.25) |  |
| Moderate injury | 188/2,917 | 2.34 | 1.24 (1.07-1.44) |  |
| Severe injury | 58/768 | 2.81 | 1.59 (1.22-2.06) |  |
| Overall |  |  | 1.19 (1.10-1.28) |  |

Abbreviations: MI, myocardial infarction; PYrs, person-years; HR, hazard ratio; CI, confidence interval **Table S3 Sensitivity analysis excluding injured patients who had been injured for more than 30 years at baseline (N = 59,321).**

|  | **Cases/Total** | **Incident rate/1000PYrs** | **Multivariate adjusted model** | ***P* for trend** |
| --- | --- | --- | --- | --- |
| **MI** | | | | |
| Uninjured | 657/47,034 | 0.56 | Ref. | 0.1848 |
| Mild injury | 115/9,069 | 0.48 | 0.89 (0.73-1.09) |  |
| Moderate injury | 43/2,520 | 0.69 | 1.26 (0.93-1.72) |  |
| Severe injury | 18/698 | 1.04 | 1.59 (1.00-2.55) |  |
| Overall |  |  | 1.01 (0.85-1.19) |  |
| **Stroke** | | | | |
| Uninjured | 2,623/47,034 | 2.23 | Ref. | 0.0044 |
| Mild injury | 537/9,069 | 2.27 | 1.00 (0.91-1.10) |  |
| Moderate injury | 167/2,520 | 2.71 | 1.23 (1.05-1.43) |  |
| Severe injury | 58/698 | 3.38 | 1.42 (1.09-1.84) |  |
| Overall |  |  | 1.07 (0.98-1.16) |  |

Abbreviations: MI, myocardial infarction; PYrs, person-years; HR, hazard ratio; CI, confidence interval **Table S4 Sensitivity analysis excluding injured participants who had been injured for less than 5 years (N = 57,916).**

|  | **Cases/Total** | **Incident rate/1000PYrs** | **Multivariate adjusted model** | ***P* for trend** |
| --- | --- | --- | --- | --- |
| **MI** | | | | |
| Uninjured | 710/46,798 | 0.49 | Ref. | 0.0383 |
| Mild injury | 120/8,140 | 0.45 | 0.95 (0.79-1.16) |  |
| Moderate injury | 43/2,297 | 0.59 | 1.17 (0.86-1.60) |  |
| Severe injury | 21/681 | 1.06 | 2.07 (1.34-3.20) |  |
| Overall |  |  | 1.07 (0.91-1.25) |  |
| **Stroke** | | | | |
| Uninjured | 2,713/46,798 | 1.87 | Ref. | <0.0001 |
| Mild injury | 543/8,140 | 2.07 | 1.11 (1.01-1.21) |  |
| Moderate injury | 166/2,297 | 2.28 | 1.17 (1.00-1.21) |  |
| Severe injury | 59/681 | 3.02 | 1.67 (1.29-2.16) |  |
| Overall |  |  | 1.15 (1.06-1.25) |  |

Abbreviations: MI, myocardial infarction; PYrs, person-years; HR, hazard ratio; CI, confidence interval

**Table S5 Sensitivity analysis in men subgroup (N = 66,690).**

|  | **Cases/Total** | **Incident rate/1000PYrs** | **Multivariate adjusted model** | ***P* for trend** |
| --- | --- | --- | --- | --- |
| **MI** | | | | |
| Uninjured | 800/53,352 | 0.53 | Ref. | 0.0115 |
| Mild injury | 138/9,788 | 0.49 | 0.97 (0.81-1.17) |  |
| Moderate injury | 53/2,794 | 0.68 | 1.29 (0.98-1.70) |  |
| Severe injury | 22/756 | 1.08 | 1.96 (1.28-3.01) |  |
| Overall |  |  | 1.10 (0.94-1.28) |  |
| **Stroke** | | | | |
| Uninjured | 3,084/53,352 | 2.06 | Ref. | <0.0001 |
| Mild injury | 623/9,788 | 2.24 | 1.11 (1.02-1.21) |  |
| Moderate injury | 196/2,794 | 2.54 | 1.22 (1.05-1.41) |  |
| Severe injury | 64/756 | 3.17 | 1.61 (1.25-2.06) |  |
| Overall |  |  | 1.16 (1.07-1.28) |  |

Abbreviations: MI, myocardial infarction; PYrs, person-years; HR, hazard ratio; CI, confidence interval

**Table S6 Sensitivity analysis in women subgroup (N = 3,175).**

|  | **Cases/Total** | **Incident rate/1000PYrs** | **Multivariate adjusted model** | ***P* for trend** |
| --- | --- | --- | --- | --- |
| **MI** | | | | |
| Uninjured | 15/2,540 | 0.22 | Ref. | 0.9537 |
| Mild injury | 3/471 | 0.24 | 1.16 (0.33-4.04) |  |
| Moderate injury | 1/143 | 0.26 | 1.12 (0.15-8.63) |  |
| Severe injury | 0/21 | 0 | / |  |
| Overall |  |  | 1.08 (0.36-3.26) |  |
| **Stroke** | | | | |
| Uninjured | 75/2,540 | 1.10 | Ref. | 0.2135 |
| Mild injury | 19/471 | 1.52 | 1.33 (0.80-2.21) |  |
| Moderate injury | 6/143 | 1.56 | 1.45 (0.63-3.35) |  |
| Severe injury | 1/21 | 1.58 | 1.26 (0.17-9.20) |  |
| Overall |  |  | 1.35 (0.86-2.12) |  |

Abbreviations: MI, myocardial infarction; PYrs, person-years; HR, hazard ratio; CI, confidence interval
